# A comparative evaluation on the energetic values and digestibility of fatty acids in rice bran oil and palm oil for broilers

**DOI:** 10.1016/j.psj.2022.101954

**Published:** 2022-05-06

**Authors:** Mingqiang Song, Feng Zhao, Xiaomeng Ye, Jingjing Xie, Renna Sa, Guangmin Zhang, Yuming Wang

**Affiliations:** ⁎ The State Key Laboratory of Animal Nutrition, Institute of Animal Sciences, Chinese Academy of Agricultural Sciences, Beijing 100193, China; † Beijing Challenge Group Co. Ltd., Beijing, 100081, China

**Keywords:** broiler, fatty acid digestibility, metabolizable energy, palm oil, rice bran oil

## Abstract

The objective of this study was to compare the digestibility of energy and fatty acids (**FA**) in rice bran oil (**RBO**) and palm oil (**PO**) fed to growing Arbor Acre (**AA**) broilers. A corn-soybean meal basal diet and the basal diet supplemented with 8% RBO or PO were evaluated. A total of 72 AA male broilers (initial BW = 1,173 ± 6 g; age = 22 d) were randomly divided to 3 dietary treatments with 6 replicates of 4 broilers in each. The growth performance and the ME and FA digestibility in oils were compared with a balance experiment of broilers from d 22 to 28. The ME of the RBO diet was greater (*P* < 0.05) than PO diet and basal diet, and the ME of the PO diet was greater (*P* < 0.05) than basal diet. However, no statistical difference was observed in the ME intake of broilers fed basal, RBO, and PO diets. To maintain daily ME intake, broilers ingested more basal diet relative to other diets, resulting in increased CP intake (*P* < 0.01) and retention (*P* < 0.01) than broilers fed diets supplemented with RBO and PO. This finding resulted in greater (*P* < 0.01) BWG and ADG from d 22 to 28 for broilers fed the basal diet relative to other diets, but there was no difference for BWG and ADG across oil sources. However, broilers fed RBO had numerically greater BWD and ADG than those fed PO, likely because the RBO provided greater AME, AMEn, AME/GE, AMEn/GE (*P* < 0.01) as well as ether extract (**EE**) digestibility (*P* = 0.0536) relative to PO. The digestibility of palmitic (**C16:0**), stearic (**C18:0**), oleic (**C18:1**), and linoleic (**C18:2**) were greater (*P* < 0.01) in RBO than PO, which positively influenced the energy values for RBO. These results indicate RBO has greater ME and digestibility of EE and FA, which positively influenced the growth performance of AA broilers. Therefore, RBO can be used to replace PO in broiler diets.

## INTRODUCTION

Modern broilers are faster growing than their historical counterparts but have similar feed intake; consequently, diets must be formulated with greater ME to maintain daily energy intakes. Musigwa et al. (2021) reported dietary energy accounted for approximately 75% of costs in broiler diets. Therefore, it is very important to screen low-cost lipids to reduce the production costs of broilers and compare lipid sources on a cost per unit of ME. Relative to other sources, palm oil (**PO**) is the most cost-effective lipid in the market and extensively used in poultry industry (Benzertiha et al., 2019; Saminathan et al., 2020). However, its price in the Chinese market has increased by about 40% since 2018 (Cao, 2020). Von Schaumburg et al. (2019) reported that the ME of PO was 82.5% of soybean oil (**SO**) in adult Single Comb White Leghorn roosters. However, others reported the ME of PO ranged from 5,302 to 7,231 kcal/kg for broilers (NRC, 1994; CVB, 2021), which indicates there is substantial variation of ME in PO across sources. In recent years, rice bran oil (**RBO**) has become more common in commercial diets in Brazil and China (Moraes et al., 2009; Sbardella et al., 2012; Su et al., 2015), because of increased production of low-fat rice bran (Punia et al., 2021) and reduced price than PO. The ME value of semi-refined RBO was 82.24% of purified SO for weaning pigs (Sbardella et al., 2012), but few studies have evaluated the ME of RBO for broilers. Concentration of mono unsaturated fatty acid (**MUFA**) such as oleic (**C18:1**) (Moraes et al., 2009) are greater for RBO relative to PO, but RBO has greater viscosity than PO (Punia et al., 2021). A high concentration of unsaturated fatty acid (**UFA**) is generally beneficial to the dissolution and absorption of lipids in the digestive tract positively influencing the digestibility of fatty acids (**FA**) and ME (Ravindran et al., 2016; Rodriguez-Sanchez et al., 2019), but an elevated viscosity could reduce the FA digestibility and ME of RBO. Therefore, the objective of this study was to compare ME between RBO and PO and investigate whether RBO could be an alternative to PO for broilers.

## MATERIALS AND METHODS

All experimental procedures related to the use of live broilers were approved by the animal care and welfare committee of the Institute of Animal Sciences, Chinese Academy of Agricultural Sciences (Beijing, China). The code of ethical inspection was IAS 2021-221.

### Oils and Experimental Diets

The RBO and PO were sampled from Sunner Development Inc. (Fujian, China). At 41°C, RBO was a brownish viscous liquid and PO (grade 3) was a yellow opaque liquid, and the PO has a higher viscosity than RBO (Table 1). Other chemical properties and FA composition of the RBO and PO are presented in Table 1. In the balance experiment, a corn-soybean meal diet was used as basal diet and the other two experimental diets were 92% of basal diet with 8% of RBO or PO, respectively (Table 2).

**Table 1:** Chemical characteristics and prevalent fatty acid composition of oils.

Items	Rice bran oil	Palm oil
Viscosity, mPa·s	193.3	233.7
Moisture, %	0.11	0.07
Insoluble impurities, %	0.06	0.01
Unsaponifiable matter, %	7.35	0.12
GE, kcal/kg	9,529	9,384
FFA, %	0.53	1.30
AV, mg/g	0.62	1.31
Fatty acid contents, %		
Myristic (C14:0)	0.30	0.96
Palmitic (C16:0)	17.61	48.53
Palmitoleic (C16:1)	0.15	0.12
Stearic (C18:0)	2.13	5.09
Oleic (C18:1)	34.54	33.32
Linoleic (C18:2)	28.26	6.49
Linolenic (C18:3)	1.05	0.14
Other	3.12	1.14
UFA	64.65	40.18
SFA	22.51	55.61
U:S	2.87	0.72
Iodine value^1^	81.54	40.38

1 Iodine value = (C16:1) × 0.95 + (C18:1) × 0.86 + (C18:2) × 1.732  +  (C18:3) × 2.616, which was described by Shurson et al. (2015). AV: acid value; FFA: free fatty acids; GE: gross energy; SFA: saturated fatty acids; UFA: unsaturated fatty acids; U:S: the ratio of UFA to SFA.

**Table 2:** Ingredients and chemical composition of experimental diets (air-dry basis, %).

Items	Starter and grower diet (d 1 to 21)	Balance experiment (d 22 to 28)		
		Basal diet	Rice bran oil diet	Palm oil diet
Corn	53.40	69.78	64.20	64.20
Soybean meal	35.38	25.74	23.68	23.68
Rice bran oil	–	–	8.00	
Palm oil	–	–	–	8.00
Soybean oil	4.51	–	–	–
Corn gluten meal	2.00	–	–	–
Dicalcium phosphate	1.73	1.75	1.61	1.61
Limestone	1.07	1.14	1.05	1.05
Premix^1^	0.50	0.50	0.46	0.46
Sodium chloride	0.30	0.30	0.28	0.28
L-lysine	0.50	0.35	0.32	0.32
DL-methionine	0.23	0.21	0.19	0.19
L-threonine	0.19	0.15	0.14	0.14
L-valine	0.14	0.08	0.07	0.07
Broiler complex enzyme	0.03	–	–	–
Phytase	0.02	–	–	–
Total, %	100.00	100.00	100.00	100.00
Nutrient content, %				
DM^2^	91.75	91.39	91.37	91.54
GE^2^, kcal/kg	4,714	4,309	4,765	4,751
CP^2^	24.57	19.62	17.76	18.02
EE^2^	8.21	3.44	11.96	11.96
Calcium	0.96	0.96	0.96	0.96
Total phosphorus	0.42	0.42	0.42	0.42

Abbreviation: CP, crude protein; DM, dry matter; EE, ether extract; GE, gross energy.

1 Supplied per kilogram of diet: vitamin A, 8,000 IU; vitamin D_3_, 1,000 IU; vitamin E, 20.0 IU; vitamin K_3_, 0.80 mg; thiamine, 3.0 mg; riboflavin, 8.0 mg; vitamin B_6,_ 5.0 mg; vitamin B_12_, 20.0 µg; pantothenic acid, 10.0 mg; nicotinic acid, 40.0 mg; folic acid, 0.60 mg; biotin, 0.20 mg; Cu (as copper sulfate), 8.0 mg; Fe (as ferrous sulfate), 100 mg; Mn (as manganese sulfate), 120 mg; Zn (as zinc sulfate), 100 mg; I (as calcium iodate), 0.70 mg; Se (as sodium selenite), 0.30 mg.

2 Values were determined values (DM basis), others were calculated values (air-dry basis) according to China Feed Database (2020).

### Birds Management and Experimental Design

A total of 100, one-day-old Arbor Acre (**AA**) male broiler were obtained from the hatchery and housed in 3-tier cages. From d 1 to 21, broilers were provided a corn-soybean meal diet formulated to meet the requirements of Arbor Acres Broiler Nutrition Specifications (2019), which exceeds the recommendation of nutrient requirements of broilers in China (NY/T 33-2004). Broilers had ad libitum access to feed and water throughout the experiment. A total of 72 AA male broiler (initial BW=1,173 ± 6 g; age = 22 d) were randomly divided to 3 treatments with 6 replicates of 4 broilers in each to compare the growth performance of broilers and to determine the ME and FA digestibility in oil.

### Growth Performance and ME Bioassay

Broilers were fed diets until d 28 and subsequently weighed to calculate body weight gain (**BWG**), average daily gain (**ADG**). The AME, AMEn, and FA digestibility in test oils were determined by total collection of excreta according to the technical protocol for the determination of metabolizable energy in feed for broilers recommended by Ministry of Agriculture and Rural Affairs of P. R. China. Procedures were outlined as follows: an adaption period from 09:00 on d 22 to 16:00 on d 24 (fed ad libitum experimental diets), a fast of 17 h from 16:00 on d 24 to 09:00 on d 25 to enables the complete emptying of the bird's digestive tract of feed, experimental diets were fed ad libitum starting at 09:00 on d 25 to 16:00 on d 27, and finally another fast (17 h) was started at 16:00 on d 27 to 09:00 on d 28 to collection total excreta (Bourdillon et al., 1990). Excreta from each replicated cage were collected 3 times per day through plastic trays from 09:00 on d 25 to 09:00 on d 28, and stored in an aluminum foil box at −20°C. After the collection, the excreta were transferred to an air-force drying oven at 65°C for drying.

### Chemical Analysis

Samples were ground finely with a laboratory mill (model BJ-150, Deqing Baijie Electrical Co., Ltd., Zhejiang, China) and passed through a 0.42 mm mesh screen prior to chemical analysis. The DM content was determined according to the method of AOAC (2007); the gross energy (**GE**) was measured according to the method of AOAC (2007) using a Parr 6400 automatic adiabatic oximeter (Parr instrument company, Moline, IL); the crude protein (**CP**) content was determined by the method of AOAC (2007) using a Kjeldahl nitrogen analyzer (model KDY-9820, Shandong Haineng Scientific Instruments Co., Ltd., Dezhou, China); the ether extract (**EE**) content was determined according to the method of AOAC (2007) using an automatic fat analyzer (model SOX-406, Shandong Haineng Scientific Instruments Co., Ltd., Dezhou, Shandong); the viscosity was determined according to the method of Furukawa et al. (2020). Moisture, insoluble impurities, unsaponifiable matter, free fatty acids (**FFA**) and acid value (**AV**) in lipids was carried out according to standard AOCS methods (Ca 2c-25, Ca 3a-46, Ca 6a-40, Ca 5a-40, and Cd 3d-63). The FA composition in lipids, diets and excreta was evaluated by gas chromatography (7890B series, Agilent Technologies, Wilmington, DE) with reference to the standard AOAC (2007).

### Calculation and Statistical Analysis

Dietary ME values were calculated according to the following formula:

$$\text{AME}\left(\text{kcal}/\text{kg}\text{DM}\right)=\left(GE_{d}-GE_{e}\right)/\text{FI},$$

AMEn(kcal/kgDM)=AME−(RN×8.22)/FI,

in which GE_d_ and GE_e_ are the GE intake (kcal DM) from the diet and the GE output from excreta (kcal DM), respectively; FI is the feed intake (kg DM); RN is the retained nitrogen (kg DM); 8.22 is the nitrogen correction factor (kcal/g).

The ME of oils and digestibility of EE (**DEE**) and FA were calculated according to the formula of Kong and Adeola (2014):

$$\text{AM}E_{\text{oil}}\left(\text{kcal}/\text{kg}\text{DM}\right)=\left(\text{AM}E_{\text{od}}-\text{AM}E_{\text{bd}}\times P_{\text{bd}}\right)/P_{\text{oil}}$$

$$\text{DC}\left(\%\right)=\left(DC_{\text{od}}-DC_{\text{bd}}\times \left(1-P_{\text{ti}}\right)\right)/P_{\text{ti}}$$

in which AME_bd_ is the AME (kcal/kg DM) of the basal diet; AME_od_ is the AME (kcal/kg DM) of the diet added test oil; P_bd_ is the proportion (%) of the basal diet in the experimental diet; P_oil_ is the proportion (%) of test oil in the experimental diet; DC_bd_, DC_od_, and DC are the digestibility (%) of EE or FA in the basal diet, experimental diet added test oil, and test oil, respectively; the P_ti_ is the proportional contribution (%) of EE or FA by the test oil to the experiment diet added test oil.

Summary statistics were calculated with the MEANS procedure of SAS 9.4 (SAS Inst. Inc., Cary, NC). The GLM procedure of SAS 9.4 (SAS Inst. Inc., Cary, NC) was used to analyze the differences in BWG, dietary intake, nutrient excretion and retention, nutrient digestibility, AME and AMEn among dietary treatments. Duncan's multiple comparison was used for mean separation. The AME, AMEn, AME/GE, AMEn/GE and digestibility of EE and FA in RBO and PO were compared by the TTEST procedure of SAS 9.4 (SAS Inst. Inc., Cary, NC). Significance was set at *P* < 0.05, whereas 0.05 < *P* ≤ 0.1 was considered a trend.

## RESULTS AND DISCUSSION

Feed intake was no different for broilers fed RBO diet vs PO diet, but broilers fed RBO or PO had lower (*P* = 0.0116) FI than those fed the basal diet (Table 3). The ME of the RBO diet (3,653 and 3,497 kcal/kg for AME and AMEn, respectively) was greater (*P* < 0.05) than that of PO diet (3,538 and 3,379 kcal/kg for AME and AMEn, respectively) and basal diet (3,333 and 3,165 kcal/kg for AME and AMEn, respectively). Also, the ME of the PO diet were 6.15% for AME and 6.76% for AMEn greater (*P* < 0.05) than basal diet. The ME intake did not differ across diets, but the CP concentration of basal diet was higher than that in 2 oil diets. Other researchers have also observed that feed intake of broilers decreased with increasing dietary ME concentrations (Chrystal et al., 2020). Musigwa et al. (2020) reported no difference in ME intake of broilers fed isocaloric diets with varying levels of CP, which means ME intake is independent of dietary CP. Others have established that broilers can maintain ME intake by altering feed intake to match the ME concentration within the diet within the limits of their digestive tract capacity (NRC, 1994; Musigwa et al., 2021).

**Table 3:** Nutrient balance and growth performance of AA broilers fed with basal, rice bran oil or palm oil diets.

Items	Basal diet	Rice bran oil diet	Palm oil diet	SEM	*P* value
Balance experiment (d 25 to 28)					
Feed intake, g	359.1^a^	336.2^b^	325.7^b^	7.1	0.0116
Excreta, g	91.5	85.0	86.0	2.2	0.1326
DM metabolizability, %	74.54^a^	74.69^a^	73.43^b^	0.34	0.0388
GE of diet, kcal/kg DM	4,309	4,765	4,751	–	–
GE of intake, kcal	1,547	1,602	1,543	32	0.3756
GE of excreta, kcal/kg DM	3,832^c^	4,396^b^	4,565^a^	20	<0.0001
AME, kcal/kg DM	3,333^c^	3,653^a^	3,538^b^	18	<0.0001
AMEn, kcal/kg DM	3,165^c^	3,497^a^	3,379^b^	17	<0.0001
AME of intake, kcal	1,197	1,228	1,148	25	0.1010
AMEn of intake, kcal	1,137	1,176	1,097	23	0.0865
AME/GE, %	77.35^a^	76.65^a^	74.47^b^	0.39	0.0002
AMEn/GE, %	73.46^a^	73.39^a^	71.12^b^	0.36	0.0005
CP intake, g	70.46^a^	59.72^b^	58.51^b^	1.32	<0.0001
Retention of CP, g	45.74^a^	39.71^b^	39.28^b^	0.99	0.0005
CP metabolizability, %	64.96	66.44	67.13	0.72	0.1289
EE intake, g	12.36^b^	40.20^a^	38.84^a^	0.67	<0.0001
Retention of EE, g	11.15^c^	30.97^a^	28.31^b^	0.70	<0.0001
EE digestibility, %	90.22^a^	77.01^b^	72.90^c^	1.14	<0.0001
Growth performance (d 22 to 28)					
BW on d 22, g	1,172	1,173	1,173	3	0.9960
BW on d 28, g	1,488^a^	1,454^ab^	1,436^b^	12	0.0221
BWG, g	316^a^	282^b^	263^b^	10	0.0067
ADG, g	53^a^	47^b^	44^b^	2	0.0050

a-c Within a row, means without a common superscript differ (*P*<0.05). Abbreviation: ADG, average daily gain; AME, apparent metabolizable energy; AMEn, nitrogen-corrected apparent metabolizable energy; BW, body weight; BWG, body weight gain; CP, crude protein; DM, dry matter; EE, ether extract; GE, gross energy.

Experimental diets were 92% basal diet with 8% RBO or PO. The digestibility of CP was not statistically different across diets. Broilers consumed more basal diet with greater CP, which increased the CP intake (70.46 vs. 59.72 and 58.51 g for basal, RBO and PO diets, respectively, *P* < 0.01) and retention (45.74 vs. 39.71 and 39.28 g for basal, RBO and PO diet, respectively, *P* < 0.01) relative to broilers fed diets with RBO and PO. These findings resulted in greater (*P* < 0.01) BWG and ADG from d 22 to 28 for broilers fed basal diet compared to those fed diets with RBO and PO. Accordingly, others have reported that BWG and ADG of broilers were positively correlated with the CP intake when the ME intake was identical (Musigwa et al., 2020; Sharma et al., 2021). The BWG and ADG were not different in broilers fed diets with RBO compared to PO, but higher numerical values were observed in broilers fed RBO diet relative to PO diet. This finding may relate to greater digestible fat for diet with RBO compared to PO (30.97 vs. 28.31 g, *P* < 0.05). When the AME intake was similar (*P* = 0.1010) for the 2 diets, but a greater digestible fat intake may supply more net energy. Therefore, the BWG and ADG of broilers increased with net energy intake when protein intake was similar (Zhao and Kim, 2017). The GE content of excreta in broilers fed basal diet was less (*P* < 0.05) than that of broilers fed RBO and PO diets. The GE content of excreta in broilers fed RBO diet was less (*P* < 0.05) than that of broilers fed PO diets, indicating greater undigested fat in the excreta of broilers fed PO diets. Accordingly, the EE digestibility of basal diet was greater (*P* < 0.01) than that of RBO and PO diets, and the EE digestibility of RBO diet was greater (*P* < 0.05) than that of PO diet. The RBO contains greater UFA than PO, which facilitates the absorption of FA in broilers (Rodriguez-Sanchez et al., 2019).

In the determination of ME in lipids, Aardsma and Parsons (2017) suggested that a high inclusion rate of lipid in the experimental diet may reduce its ME value by substitution. However, Vieira et al. (2015) and Von Schaumburg et al. (2019) found that up to 10% dietary PO did not affect the ME of oil. Additionally, a high inclusion of lipid would reduce the variability of ME calculated by substitution method, which is beneficial to compare the ME of lipids from different sources (Von Schaumburg et al., 2019). Thus, the inclusion rate of 8% for oils in the experimental diets is appropriate in the current study. The AME (6,908 vs. 5,628 kcal/kg, *P* = 0.0020), AMEn (6,879 vs. 5,556 kcal/kg, *P* = 0.0012), AME/GE (72.50 vs. 59.98%, *P* = 0.0033), AMEn/GE (72.19 vs. 59.21%, *P* = 0.0021) and DEE (72.38 vs. 66.82%, *P* = 0.0536) were greater for RBO compared to PO (Table 4). The AME of the diet with RBO was 103.2% that of PO. This finding agreed with results reported by Ruan et al. (2013), who found that the G/F of RBO diet was 101.03% of PO diet when oils were supplemented at 2.5 and 4% of inclusion rate in diets fed broilers from d 0 to 21 and d 22 to 42, respectively. Similar to the observation of Ruan et al. (2013), the current RBO contained less stearic (**C18:0**) but more linoleic (**C18:2**) than PO, which led to the energetic values of RBO was greater than PO. High ratio of UFA to saturated fatty acids (**SFA**) increases the digestibility of lipid (Wiseman and Salvador, 1991). Therefore, the ME of RBO and its efficacy on the growth of broilers was greater than PO.

**Table 4:** Energetic values and digestibility of fatty acid of rice bran oil and palm oil fed to AA broilers.

Items	Rice bran oil	Palm oil	SEM	*P* value
AME, kcal/kg	6,908	5,628	308	0.0020
AMEn, kcal/kg	6,879	5,556	297	0.0012
AME/GE, %	72.50	59.98	3.27	0.0033
AMEn/GE, %	72.19	59.21	3.15	0.0021
EE digestibility, %	72.38	66.82	2.54	0.0536
Digestibility of fatty acid, %				
Myristic (C14:0)	88.50	71.30	1.40	<0.0001
Palmitic (C16:0)	79.55	54.58	3.56	<0.0001
Palmitoleic (C16:1)	90.91	81.65	2.14	0.0015
Stearic (C18:0)	68.76	44.49	4.55	0.0003
Oleic (C18:1)	88.60	82.84	2.17	0.0242
Linoleic (C18:2)	88.00	70.69	3.86	0.0012
Linolenic (C18:3)	89.14	64.24	5.02	0.0026

AME: apparent metabolizable energy; AMEn: nitrogen-corrected apparent metabolizable energy; EE: ether extract; GE: gross energy.

The digestibilities of palmitic (**C16:0**) (79.55 vs. 54.58%, *P* < 0.01), C18:0 (68.76 vs. 44.49%, *P* = 0.0003), C18:1 (88.60 vs. 82.84%, *P* = 0.0242) and C18:2 (88.00 vs. 70.69%, *P* = 0.0012) were greater in RBO than PO (Table 4), which contributed to the greater energetic value for RBO than PO. In the current study, broilers fed RBO diet consumed more UFA than PO diet, which may lead to the higher digestibility of SFA in RBO than PO. Others have reported that the digestibility of SFA is increased with higher concentration of UFA (Crespo and Esteve-Garcia, 2002). The AME (5,628 kcal/kg) and DEE (66.82%) of the current PO were less than that or PO described by Tancharoenrat et al. (2013) (8,281 kcal/kg and 83.6% for DEE) and Vieira et al. (2015) (8,884 kcal/kg for AME and 80.11% for DEE), but similar to the AME of PO in the Chinese feed database (5,800 kcal/kg for AME; Xiong et al., 2020). These divergent findings may relate to greater C16:0 and reduced C18:1 content for the current PO relative to that used in the studies by Tancharoenrat et al. (2013) and Vieira et al. (2015). However, the current PO was relatively similar in content of C16:0 and C18:1 compared to PO described in the Chinese feed database (Xiong et al., 2020). The digestibility of SFA is generally less than UFA (Ravindran et al., 2016). Therefore, the SFA content in the current PO relative to others reported in the literature resulted in a relatively low ME. Those findings indicate that it is important to understand the content of prevailing FAs such as C16:0 and C18:1, when establishing ME values of dietary lipids.

## CONCLUSIONS

This experiment showed that RBO had greater AME/GE, AMEn/GE, and digestibility of EE and FAs, AME, AMEn than that of PO in AA broilers. Therefore, RBO can be used to replace PO in broiler diet. Additionally, when assessing the AME of lipid sources, the concentration of UFA should be considered. Prediction equations for the AME of RBO and PO from FA composition should be established.
